# Time-dependent effects of late-onset dietary intake of salidroside on lifespan and age-related biomarkers of the annual fish *Nothobranchius guentheri*

**DOI:** 10.18632/oncotarget.23957

**Published:** 2018-01-04

**Authors:** Xia Wang, Xiaoyuan Du, Yang Zhou, Su Wang, Feng Su, Shicui Zhang

**Affiliations:** ^1^ Institute of Evolution and Marine Biodiversity and Department of Marine Biology, Ocean University of China, Qingdao 266003, China; ^2^ Laboratory for Marine Biology and Biotechnology, Qingdao National Laboratory for Marine Science and Technology, Qingdao 266003, China; ^3^ Institute of Chemical Engineering, Qingdao University of Science and Technology, Qingdao 266042, China

**Keywords:** salidroside, aging, lifespan, antioxidant system, Nothobranchius

## Abstract

One of the most studied and widely accepted conjectures of aging process is the oxidative stress theory. Previous studies have shown that salidroside can protect D-galactose-induced mouse model against aging and a formulation of *Rhodiola rosea* extracts (SHR-5) containing salidroside increases lifespan of fruit fly. However, direct evidence linking salidroside itself with the observed anti-aging effect *in vivo* and relevant molecular mechanisms are poorly defined. In this study, we first demonstrated that salidroside exhibited a time-dependent effect, and late-onset long-term salidroside dietary intake extended the lifespan in the annual fish *Nothobranchius guentheri*. We then showed that salidroside reduced the accumulation of lipofuscin in the gills as well as the levels of protein oxidation, lipid peroxidation and reactive oxygen species in the muscles; enhanced the activities of catalase, glutathione peroxidase, and superoxide dismutase in the fish; and decelerated the increase of P66shc, a critical factor for regulation of intracellular reactive oxygen species contents. Collectively, these data indicate that salidroside can prolong the lifespan and retard the onset of age-related biomarkers via the antioxidant system in aging fish. It also suggests that salidroside may have a potential usefulness in prolonging the lifespan of the elderly.

## INTRODUCTION

Aging is the process of becoming older, which is exemplified by the progressive physiological changes in an organism that lead to a decline of biological functions and of the organism’s ability to adapt to metabolic stress. One of the most studied and widely accepted conjectures on the molecular basis of aging process is the oxidative stress theory initially proposed by Denham Harman [1]. Oxidative stress, an imbalance in the production and detoxification of reactive oxygen species (ROS), the by-products of oxidative phosphorylation, causes damage to the macromolecules including lipids, proteins and DNA, and the organelles such as mitochondrion, thereby impairing cellular integrity and functionality, eventually resulting in progressive aging of organisms [2–5]. The theory has gained strong support from many studies that linked oxidative stress to longevity using model organisms including yeasts, flies, nematodes, and rodents [2, 6–8]. Under this mechanistic framework, down-regulation of oxidative stress may be directly beneficial to the extension of health span and lifespan. Actually, numerous studies have proved that ingestion of ROS scavenger compounds, such as resveratrol, vitamin E, and ethosuximide, prolongs an organism’s lifespan [9–13].

Salidroside (SDS), a phenylpropanoid glycoside, is a potent antioxidant component isolated from the roseroot *Rhodiola rosea*, which grows at high altitudes (up to 2280 m) in the Arctic and mountainous regions throughout Europe and Asia, and has long been used as traditional medicine in China and Eastern Europe. The main effects of SDS described include anti-hypoxia, anti-inflammatory, anti-viral, anti-cancer, anti-fatigue, immune-boosting, hepatoprotective and neuroprotective activities [14–25]. Additional studies have also proved that SDS protects D-galactose-induced mouse model against aging [18] and a formulation of *R. rosea* extracts (SHR-5) containing SDS increases both mean and maximum lifespan of fruit fly [26]. However, direct evidence linking SDS itself with the observed anti-aging effect *in vivo* and relevant molecular mechanisms are still poorly defined.

Small annual fishes, especially the genus *Nothobranchius*, have many anatomical and histological characteristics similar to those of mammalian species and a relatively short lifespan, are commercially available, and are easily reared in captivity. Thus, they have become an emerging model organism for aging studies in recent years. For example, it has been demonstrated that senescence-associated β-galactosidase, accumulation of lipofuscin (LF), levels of lipid peroxidation and protein oxidation increased with age, whereas the activities of anti-oxidant enzymes catalase (CAT), glutathione peroxidase (GPX), and superoxide dismutase (SOD) decreased with age in *N. rachovii* [27]. Additionally, resveratrol was found to be able to extend both the mean and maximum lifespans, to attenuate the increase of reactive oxygen species (ROS) and the degree of oxidative damage by up-regulating activities of anti-oxidant enzymes CAT, GPX and SOD and to preserve glia in *N. guentheri* [11, 28]. Moreover, vertebrate aging-related genes *p66Shc* and microsomal triglyceride transfer protein (MTP) could be easily isolated by homology cloning in *N. furzeri* [29]. In this study, we thus used the annual fish *Nothobranchius guentheri* as model to explore if dietary intake of SDS has any influence on aging kinetics by determination of several age-related biomarkers such as antioxidant systems, and if so, to illustrate the possibly associated molecular mechanisms.

## RESULTS

### SDS has little effects on body weight and length

Totally, 190 of 9-month-old male *N. guentheri* were divided into two groups, and fed with the SDS-containing and control diets, respectively. All the fish survived well, and little difference was observed in the behavior and locomotive activity between the fishes in the two groups throughout the experimental periods.

**Figure 1:**
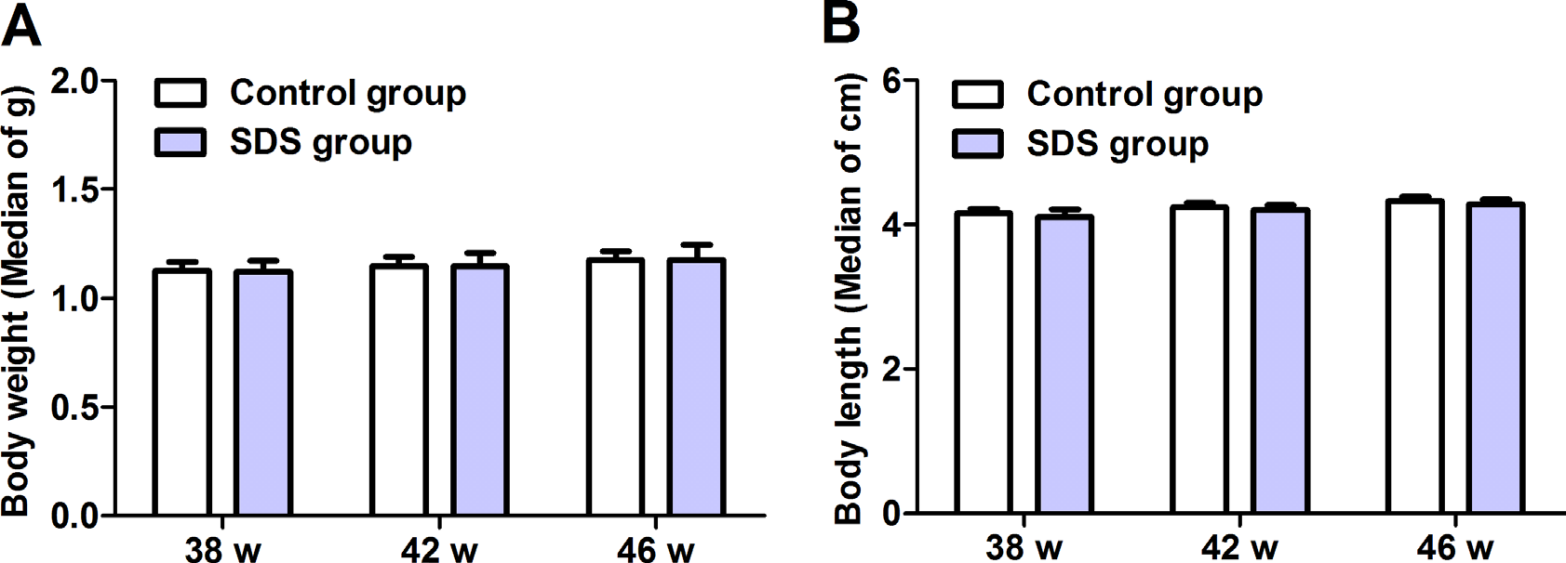
Body weight (**A**), body length (**B**) of male *N. guentheri*. The values of body weight are median of g ± standard deviation (SD) (*n* = 4), and the values of body length are median of cm ± standard deviation (SD) (*n* = 4). The data are from 4 independent experiments which were performed in triplicate. w, week.

Both the body weight and length were assessed on 38th, 42th and 46th weeks. Compared with control group, no significant differences were observed in the body weight and length of the fish in SDS group (Figure 1A, 1B; *p* > 0.05), consistent with the observation of Lu et al. [17]. These show that SDS administration has little influence on the body weight and length of aging *N. guentheri*.

### SDS prolongs lifespan

**Figure 2:**
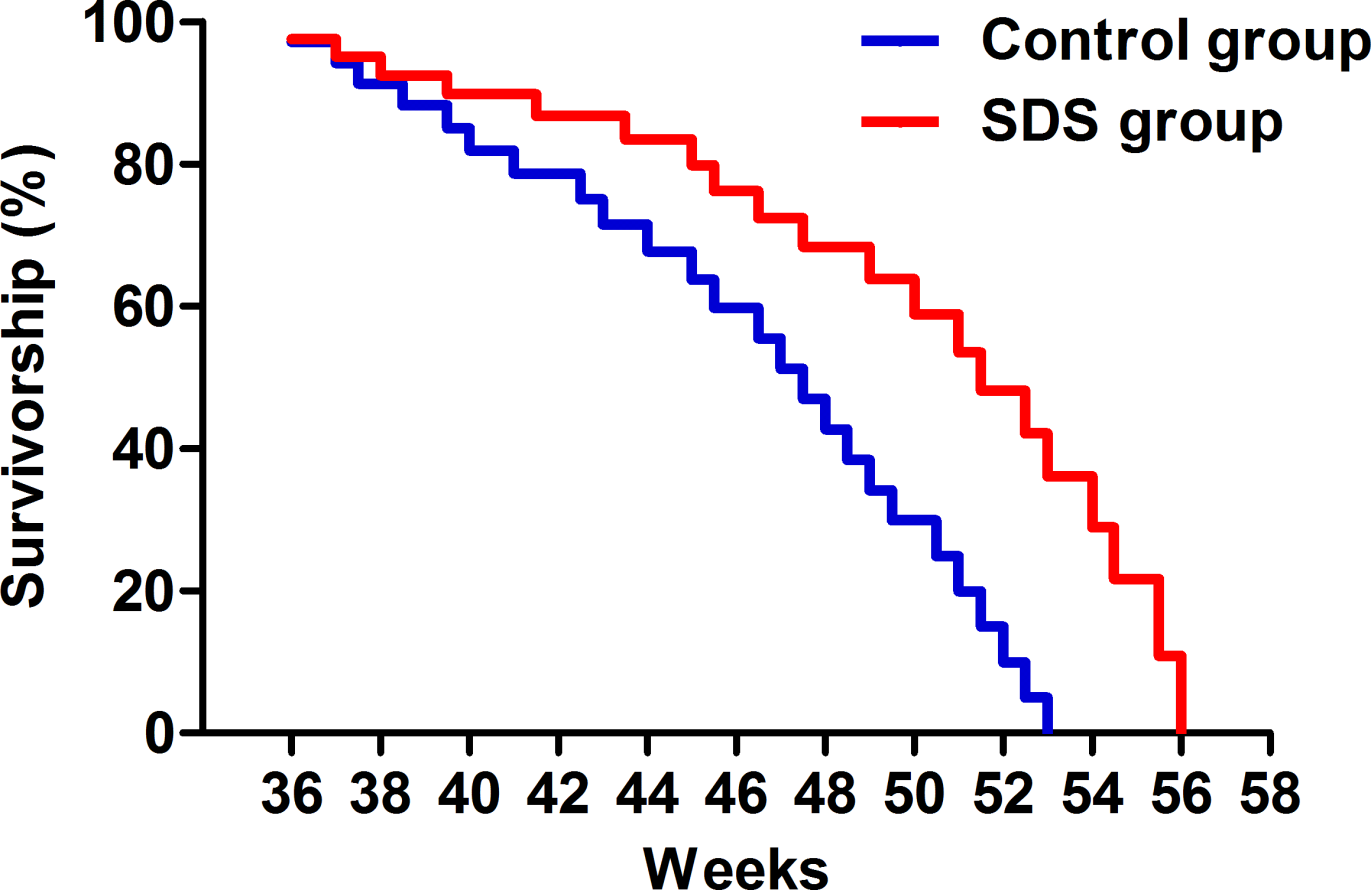
Survivorship curve The survivorship curves of male *N. guentheri* in control (*n* = 50; blue line) and SDS groups (*n* = 50; red line) (*p* < 0.05).

Survivorship curves for 9-month-old (36 weeks) male *N. guentheri* of the control and SDS groups showed that the mean lifespans of the fishes in SDS and control groups were 51.5 ± 1.3 and 47.5 ± 1.6 weeks (*p* < 0.05; Figure 2), respectively. In accordance, the maximum lifespans of the fishes in the two groups were 56 ± 1.2 and 53 ± 1.4 weeks (*p* < 0.05), individually. Obviously, SDS extended the mean and maximum lifespans of the fishes by approximately 4 and 3 weeks, separately. Therefore, late-onset SDS administration is able to prolong the survivorship of male *N. guentheri*.

### SDS reduces accumulation of LF in gills

**Figure 3:**
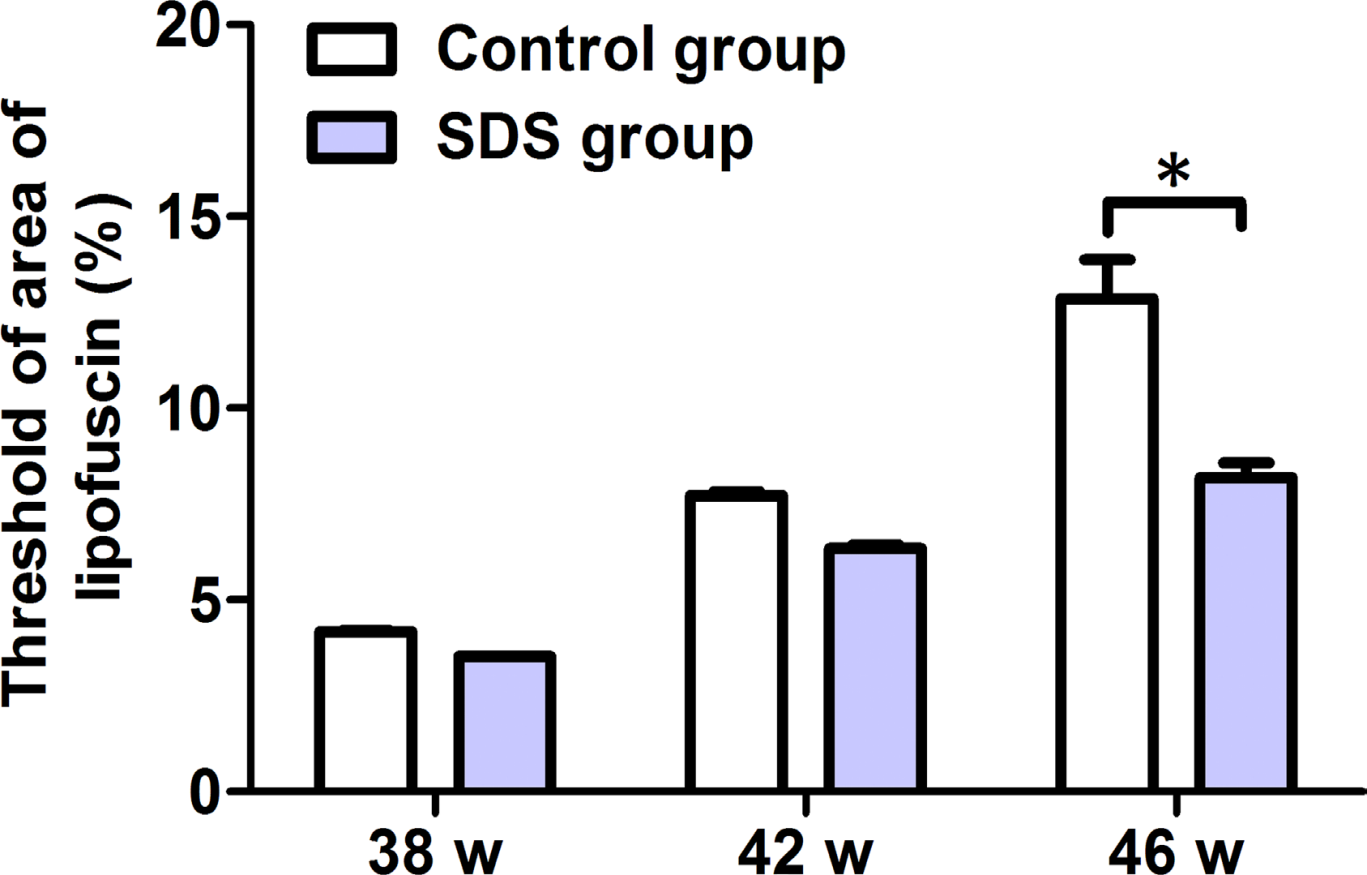
Changes of the histological marker LF in 38-, 42- and 46-week-old *N. guentheri* Statistical analysis of the threshold of areas occupied by LF in the gills of *N. guentheri* according to Supplementary Figure 3 (*n* = 4). The data are from 4 independent experiments which were performed in triplicate. The symbol (^*^) means significantly different (*p* < 0.05). w, week.

LF is visualized as bright green-colored auto-fluorescent dots in the gill cells. As shown in Figure 3 and Supplementary Table 1, SDS administration for 7 weeks (from 36th to 42th week) resulted in little difference in the green-colored areas in the gills of the fish, compared with control group (3.525 ± 0.0118% vs 4.168 ± 0.0701%; 6.348 ± 0.1563% vs 7.718 ± 0.1846%; *p* > 0.05), but SDS administration for 10 weeks (from 36th to 46th week) caused significant reduction (8.19 ± 0.6601% vs 12.86 ± 1.765%; *p* < 0.05) in the fluorescent areas in the gills of the fishes of SDS group than control group. These indicate that the effects of SDS are time-dependent, and longer term SDS administration (e.g. 10 weeks) reduces the accumulation of LF in the gills of aging *N. guentheri*.

### SDS reduces levels of protein oxidation and lipid peroxidation

**Figure 4:**
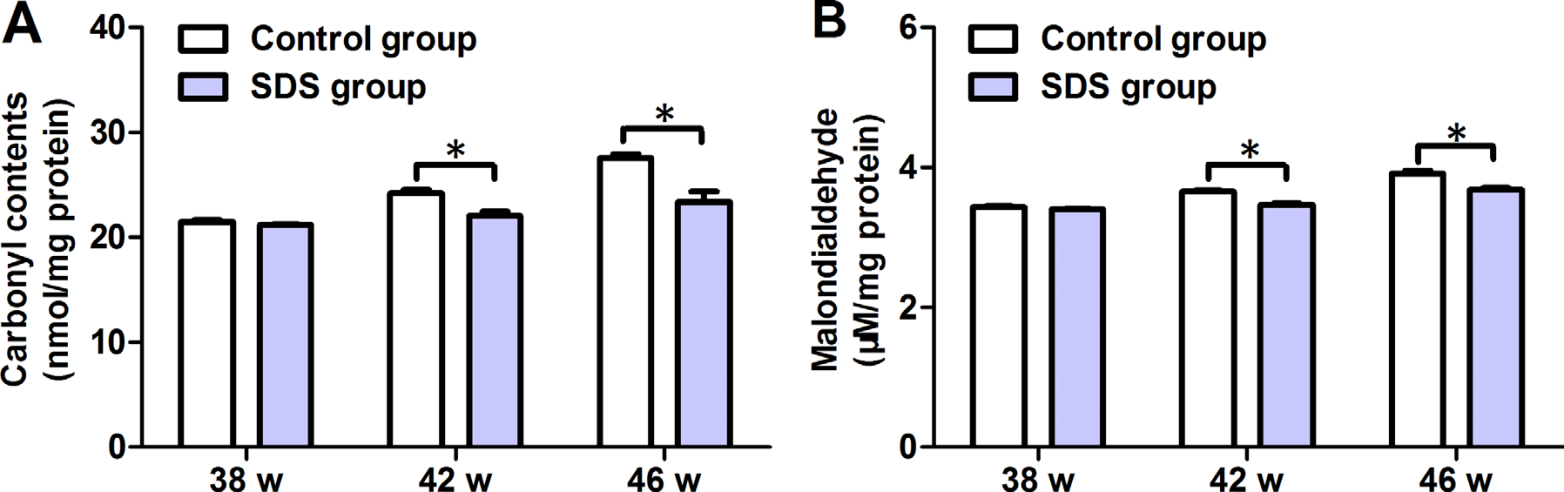
The levels of protein oxidation and lipid peroxidation in 38-, 42- and 46-week-old *N. guentheri* in control and SDS groups (**A**) Protein oxidation levels; (**B**) Lipid peroxidation levels. Data represent mean ± standard deviation (SD) (*n* = 4). The data are from 4 independent experiments which were performed in triplicate. The symbol (^*^) means significantly different (*p* < 0.05). w, week.

The contents of carbonyl-group (protein oxidation marker) and malondialdehyde (MDA, a metabolite of lipid peroxidation) acquired by the muscles were detected on 38th, 42th and 46th weeks. Little differences were found between the mean values of carbonyl-group content in the muscles of the fishes of SDS group (21.17 ± 0.137 nmol/mg protein) and control group (21.49 ± 0.318 nmol/mg protein; *p* > 0.05; Figure 4A) on 38th week, while the mean values of carbonyl-group content in the muscles of the fish of SDS group were considerably decreased on 42th and 46th weeks, compared with control group (22.04 ± 0.710 vs 24.20 ± 0.548, 23.37 ± 1.763 vs 27.58 ± 0.633 nmol/mg protein; *p* < 0.05; Figure 4A). Similarly, on 38th week, the mean values of MDA in the muscles of the fish of SDS group (3.401 ± 0.014 μM/mg protein) were not different from those in the muscles of the fish of control group (3.431 ± 0.038 μM/mg protein; *p* > 0.05; Figure 4B), whereas the mean values of MDA in the muscles of the fish of SDS group were markedly reduced on 42th and 46th week, compared with control group (3.469 ± 0.032 vs 3.654 ± 0.034, and 3.677 ± 0.0619 vs 3.919 ± 0.056 μM/mg protein; *p* < 0.05; Figure 4B). These show again that the effects of SDS are time-dependent, and longer term SDS administration reduces the levels of both carbonyl-group and MDA in the muscles of aging *N. guentheri*.

### SDS prevents decrease in CAT, GPX and SOD activities

**Figure 5:**
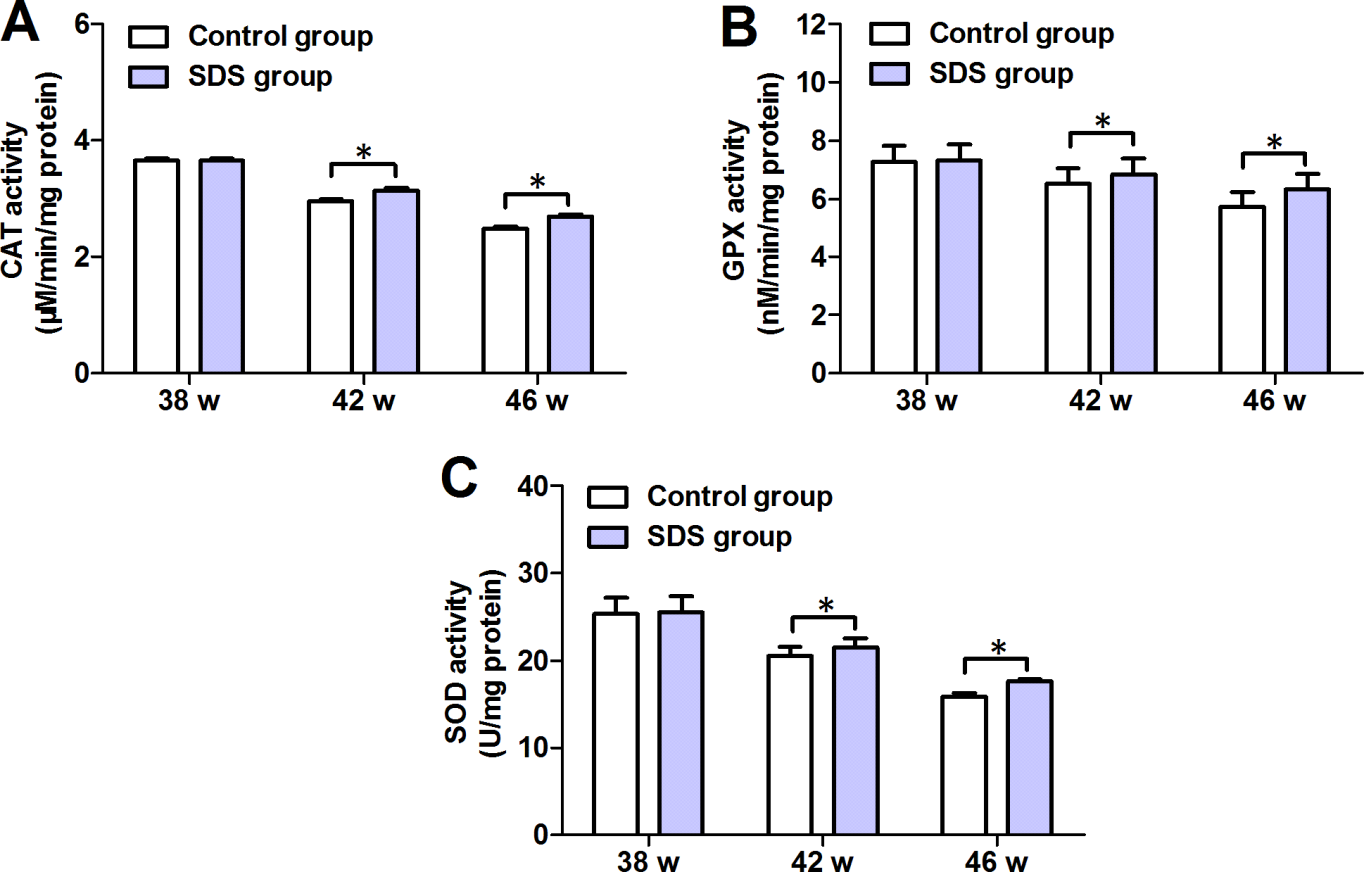
Changes in activities of CAT, GPX and SOD in 38-, 42- and 46-week-old *N. guentheri* in control and SDS groups (**A**) CAT activities; (**B**) GPX activities; (**C**) SOD activities. Data represent mean ± standard deviation (SD) (*n* = 4). The data are from 4 independent experiments which were performed in triplicate. The symbol (^*^) means significantly different (*p* < 0.05). w, week.

Figure 5 shows the changes in the activities of antioxidant enzymes CAT, GPX and SOD. On 38th week, the mean values of CAT, GPX and SOD activities in the muscles of the fish of SDS group were closely similar to those in the muscles of control fish (*p* > 0.05). By contrast, on 42th and 46th weeks, the mean values of CAT, GPX and SOD activities in the muscles of the fish of SDS group were significantly increased, compared with control group (for CAT: 3.133 ± 0.101 vs 2.950 ± 0.077 and 2.698 ± 0.052 vs 2.481 ± 0.065 μM/min/mg protein; for GPX: 6.836 ± 0.929 vs 6.529 ± 0.907 and 6.320 ± 0.950 vs 5.735 ± 0.891 nM/min/mg protein; for SOD: 21.48 ± 1.847 vs 20.53 ± 1.797 and 17.69 ± 0.227 vs 15.89 ± 0.140 U/mg protein; *p* < 0.05). These indicate that longer term but not shorter term (e.g. 3 weeks) SDS administration prevents the decrease in the activities of the anti-oxidant enzymes.

### SDS down-regulates expression of p66shc

Intracellular ROS contents can be increased by three main mechanisms: reducing ROS scavenging, increasing membrane oxidases activity, or by mitochondrial respiratory chain leakage; and P66SHC has been shown to act through all the three mechanisms. Therefore, *N. guentheri* P66shc cDNA was cloned and qRT-PCR was used to examine the expression profiles of *p66shc* in the fish. *N. guentheri* P66shc cDNA sequence we obtained was about 1785 bp, coding for a protein of 595 amino acids with a molecular mass of about 64 kDa (Supplementary Figure 2). *N. guentheri* P66shc, like mammalian P66SHC, both had four conserved domains CH2, PTB, CH1 and SH2, three highly conserved S residues and a conserved cytochrome c binding domain consisting of E, D and W residues (Supplementary Figure 3). Sequence alignment showed that *N. guentheri* P66shc was 70% to 71.5% identical to mammalian P66SHC at amino acid levels (Supplementary Figure 4).

**Figure 6:**
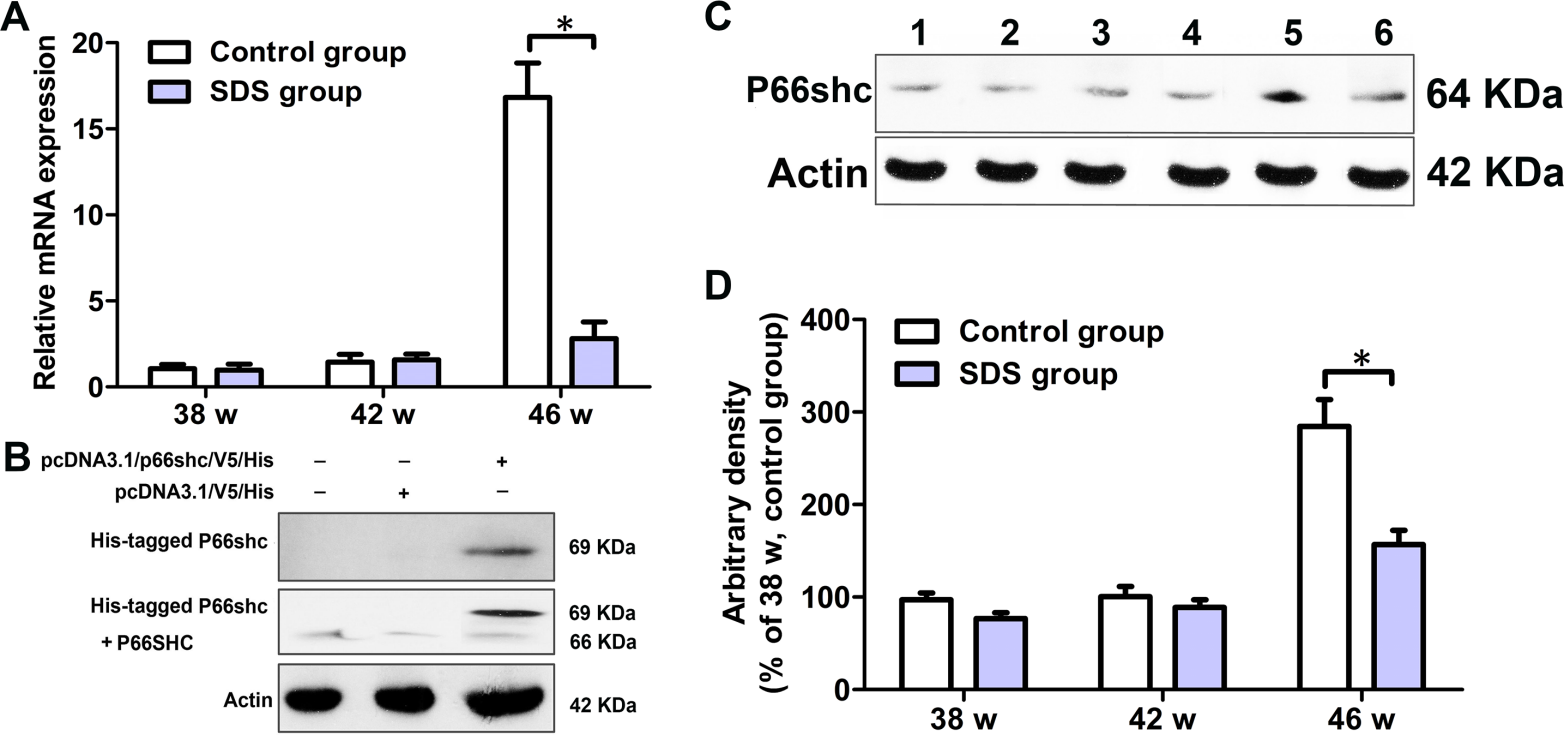
Levels of *p66shc* in the muscles of 38-, 42- and 46-week-old *N. guentheri* in control and SDS groups (**A**) Expression profile of *p66shc*. *β-actin* gene was chosen as the internal control for normalization. Relative expression data were calculated by the method of 2^-△△Ct^; (**B**) Western blotting of P66SHC in HEK 293T cells; (**C**) Western blotting of P66shc in muscles of *N. guentheri*. Lanes 1, 3 and 5, protein extracts from 38, 42 and 46-week-old *N. guentheri* muscles in control group; Lanes 2, 4 and 6, protein extracts from 38, 42 and 46-week-old *N. guentheri* muscles in SDS group; (**D**) Expression of P66shc in *N. guentheri* muscles were normalized to expression in 38-week-old *N. guentheri* muscles in control group. β-Actin was chosen as the internal control for normalization. Data represent mean ± standard deviation (SD) (n = 4). The data are from 4 independent experiments which were performed in triplicate. The symbol (^*^) means significantly different (*p* < 0.05). w, week.

The dissociation curve of amplified product showed a single peak, indicating that the amplification was specific (data not shown). On 38th and 42th weeks, mRNA levels of *p66shc* in the muscles of the fish of SDS group were similar to those in the muscles of control fish (*p* > 0.05). By contrast, on 46th week, mRNA content of *p66shc* in the muscles of the fish of SDS group was remarkably decreased, compared with control group (*p* < 0.05; Figure 6A). These results were also confirmed by Western blotting analysis. To obtain a positive control, His-tagged *N. guentheri* P66shc was first expressed in HEK 293T cells. As shown in Figure 6B (the entire gels of Western blotting were shown in Supplementary Figure 5), the extracted proteins of HEK 293T cells with *pcDNA3.1/V5/p66shc/His* reacted with anti-His tag antibody as well as with anti-SHC antibody, both producing a main band of about 69 kDa. These indicated that anti-SHC antibody was specific to P66shc. Next, we used the anti-SHC antibody to detect the expression of P66shc in *N. guentheri* muscles, and found that only a single band of about 64 kDa was visualized, which apparently represents *N. guentheri* P66shc (Figure 6C and 6D; the entire gels of Western blotting were shown in Supplementary Figure 6). Notably, no significant differences were observed between the contents of P66shc in the muscles of the fishes of SDS and control groups (*p* > 0.05) on 38th and 42th weeks, but on 46th week, the P66shc level in the muscles of the fish of SDS group was markedly decreased, compared with control group (*p* < 0.05; Figure 6C and 6D). These show that longer term SDS administration accelerates the decrease in P66shc in aging *N. guentheri*.

### SDS reduces level of ROS

**Figure 7:**
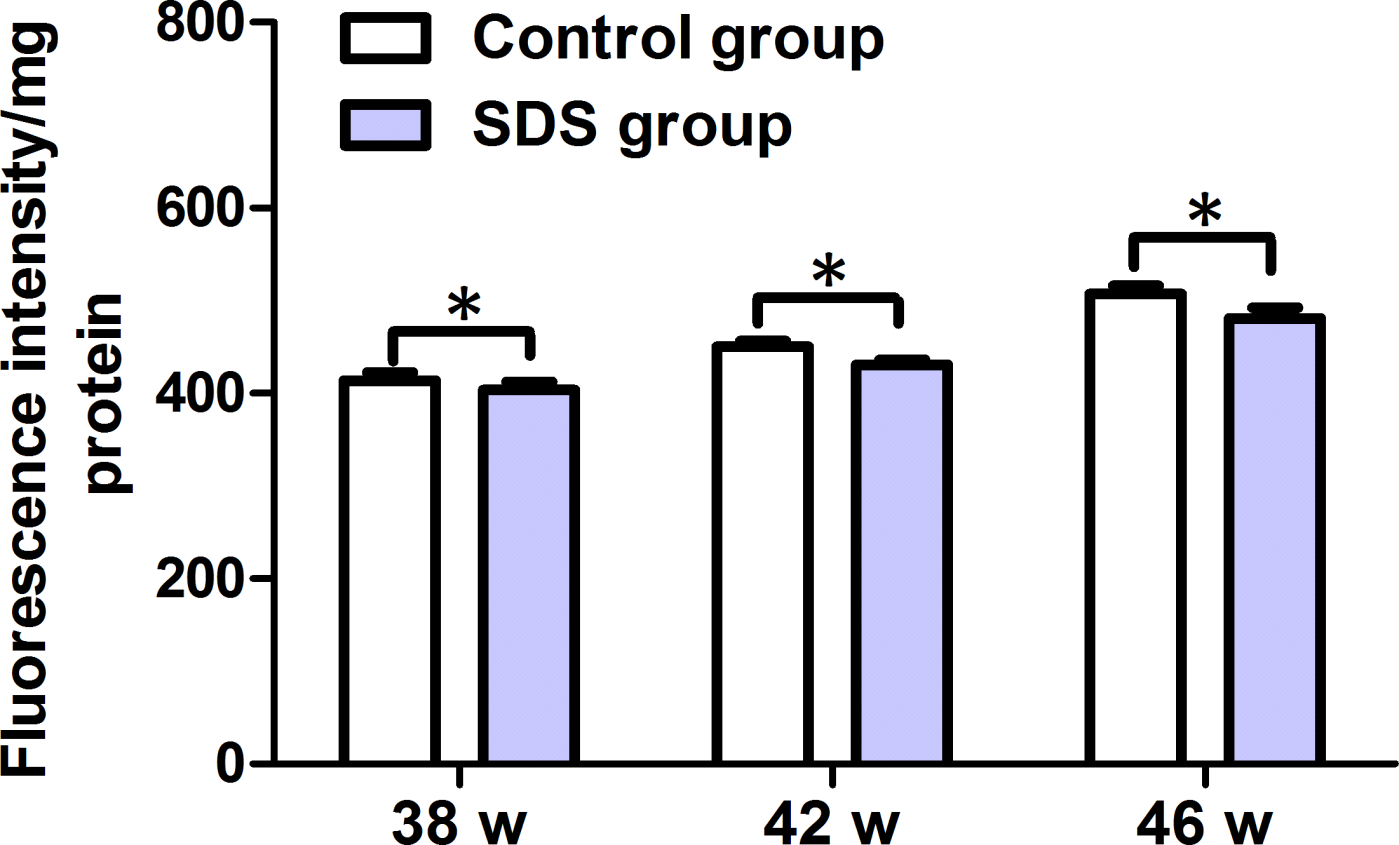
ROS levels in the muscles of 38-, 42- and 46-week-old *N. guentheri* in control and SDS groups Data represent mean ± standard deviation (SD) (*n* = 4). The data are from 4 independent experiments which were performed in triplicate. The symbol (^*^) means significantly different (*p* < 0.05). w, week.

Next, we measured the levels of ROS in the muscles on 38th, 42th and 46th weeks. As shown in Figure 7, the levels of ROS in the muscles of the fish of control group were 413.7 ± 15.32, 450.4 ± 10.05, and 506.9 ± 15.38 fluorescence intensity/mg protein on 38th, 42th and 46th weeks, respectively. By contrast, the levels of ROS in the muscles of the fish of SDS group were 403.7 ± 15.23, 430.4 ± 10.0 and 480.4 ± 20 fluorescence intensity/mg protein on 38th, 42th and 46th weeks, individually. It was clear that ROS levels decreased significantly in the muscles of the fish in SDS group than control group on 38th, 42th and 46th weeks (*p* < 0.05). These show that SDS decreases ROS levels in the muscles of aging *N. guentheri*.

## DISCUSSION

**Figure 8:**
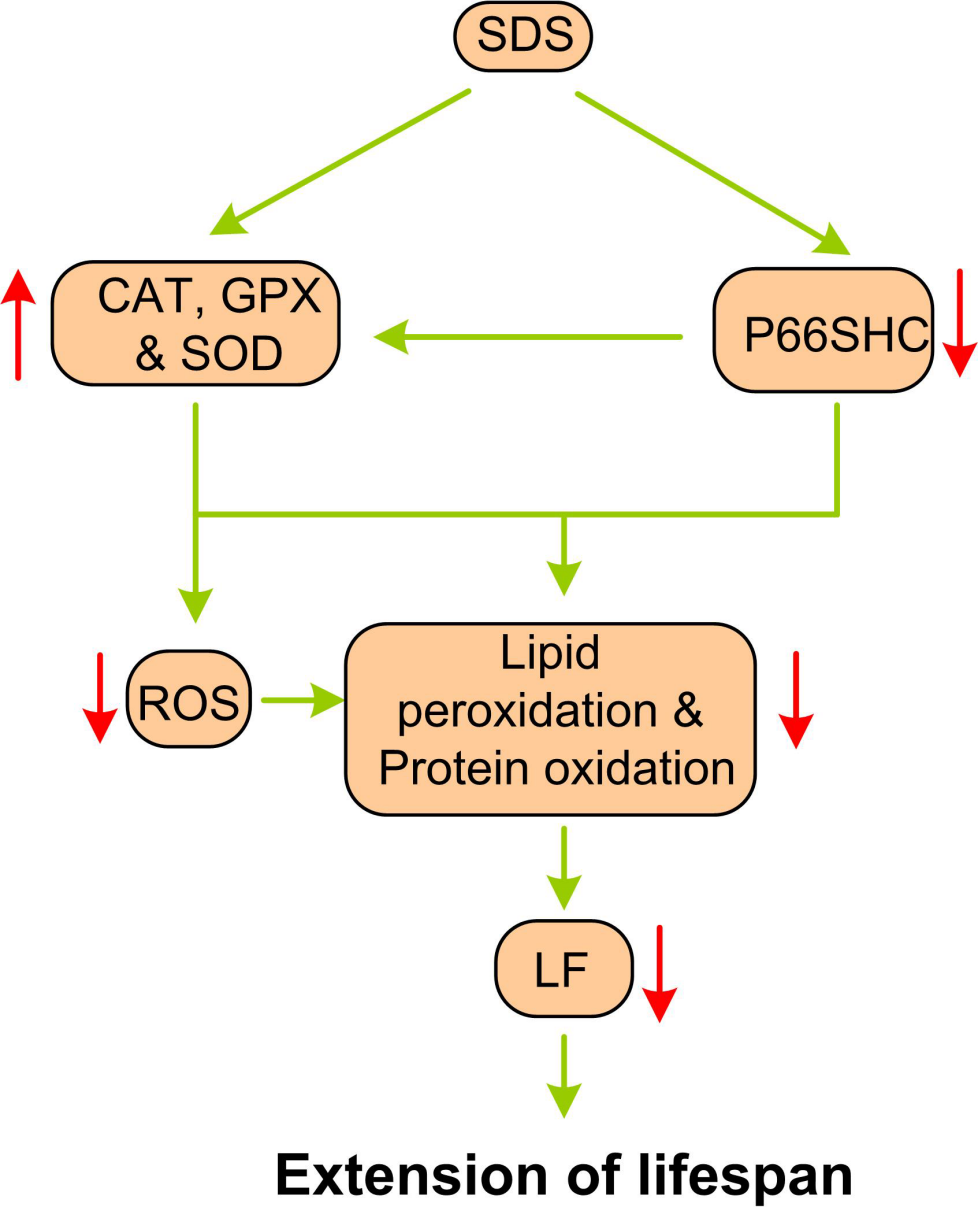
A schematic diagram showing the potential mechanism of SDS administration on aging The direction of arrows indicates the changes of biomarkers.

The phenotype of an organism, which includes characteristics such as lifespan, is determined in part by the nucleotide sequence of its genes and in part by the regulation of these genes, which is often influenced by the genome as well as the environment. Therefore, non-intrusion interventions, such as calorie restriction (CR) and temperature reduction (TR), as well as nutritional interventions with essential nutrients or functional foods, are also identified as possible approaches for prolonging an organism’s life span. For example, dietary supplementation with nutrients with antioxidant properties, such as β-carotene, vitamin E, and lycopene, has been shown to improve immune function in aged mice and humans [30–32]. Dietary intake of SDS has been shown to protect PC12 cells against H_2_O_2_-induced apoptosis [33] and HaCAT cells against UVB-mediated oxidative damage [34]. SDS has also been reported to alleviate oxidative stress in the liver with non-alcoholic steatohepatitis in rats [35], to prevent kainic acid-induced status epilepticus via suppressing oxidative stress and diabetes-induced oxidative stress in mice [36, 37], and to protect mitochondria against exertional heat stroke-induced organ damage and retinal endothelial cells against hydrogen peroxide-induced injury via modulating oxidative status and apoptosis in rats [38, 39]. The main findings of our study are that late-onset SDS administration does not reduce the body weight and length of aging *N. guentheri*, but it is able to decrease the age-related markers and increase the median and maximum lifespans of the fish. SDS appears to perform this anti-aging activity via action of antioxidant system. Our results clearly demonstrate that SDS can enhance the activities of the anti-oxidant enzymes, and reduce the level of P66shc, a potent factor responsible for regulation of intracellular ROS contents. Both the enhanced antioxidant enzyme activities and reduced P66shc level may directly lead to the reduction of ROS levels (Figure 8), which in turn slow down the protein oxidation, lipid peroxidation and possibly LF development, and eventually prolong the lifespan of aging fish.

SDS has been highly valued for many years for its anti-hypoxia, health-promoting, immune-boosting and anti-aging abilities, but studies to evaluate the time course of SDS effects remains lacking. An interesting finding of this study is that the effects of SDS are time-dependent. We demonstrate here that dietary intake of SDS for 3 weeks (from 36th to 38th week) has little effects on the parameters examined, including LF, lipid peroxidation, protein oxidation, CAT, GPX, SOD, and P66shc, but 10 week SDS administration (from 36th to 46th week) resulted in a marked decrease in the levels of LF, lipid peroxidation, protein oxidation, P66Sshc and ROS as well as a remarkable increase in the activities of antioxidant enzymes CAT, GPX and SOD. These suggest that SDS may prolong the lifespan of aging *N. guentheri* in a time-dependent fashion, and relatively longer term dietary intake is necessary for lifespan extension.

In summary, this study highlights the anti-aging property of SDS and its potential usefulness in prolonging the lifespan of aging fish (and possibly the elderly). It also shows for the first time that the anti-aging effects of SDS are time-dependent.

## MATERIALS AND METHODS

### Fish culture and diet

All the fish *N. guentheri* used in the experiments were treated in accordance with the guidelines of the Laboratory Animal Administration Law of China, with the permit number SD2007695 approved by the Ethics Committee of the Laboratory Animal Administration of Shandong province. The fish *N. guentheri* were bred in our own laboratory. It has a relatively stable lifespan. We have shown a complete survival curve of *N. guentheri* produced in our lab in Supplementary Figure 7. All of the fish were reared at a density of 5 fishes per 10-liter tank under an ambient photoperiod at 26 ± 1°C, and fed with live blood worms twice a day. As the male *N. guentheri* have median survival of approximately 12 months [40], thus the 9-month-old (i.e. 36th week) male fish were selected as model of aging fish in order to test the effect of SDS on aging process in this study.

**Figure 9:**
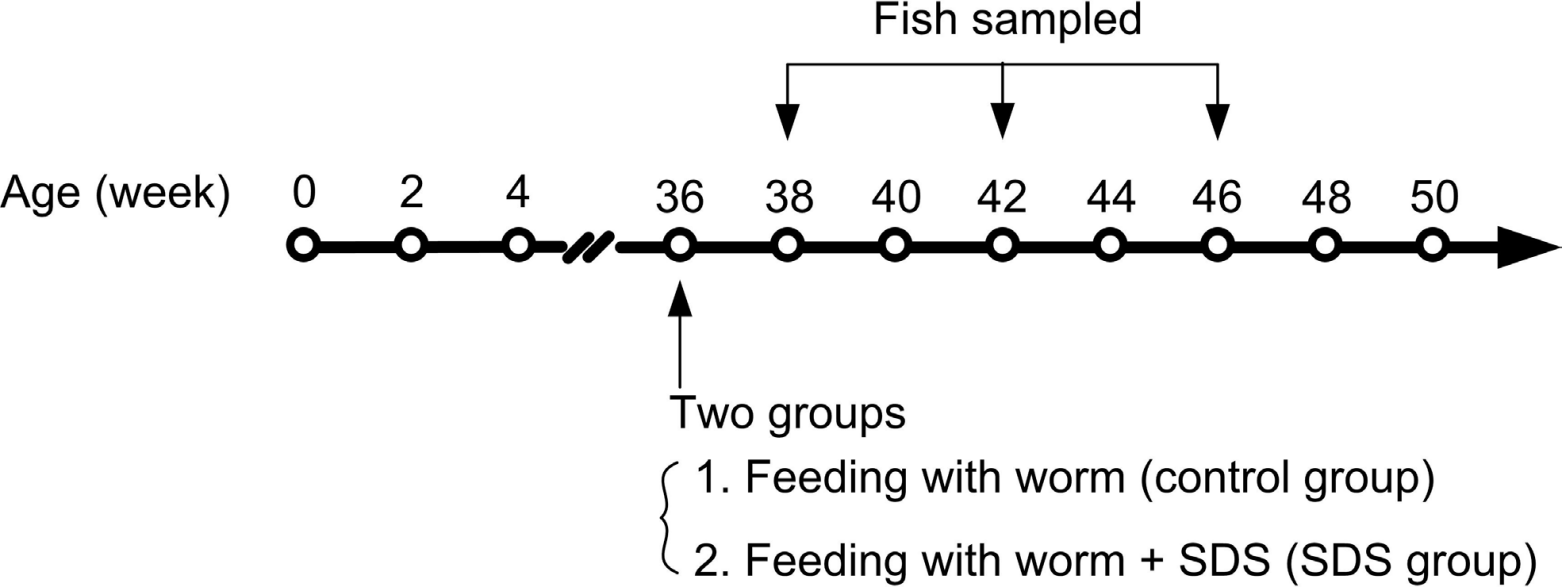
A schematic diagram of the experiments The arrows indicate time points of feeding and sampling. SDS administration began with 36th week, and four fish were sampled from each group on 38th, 42th and 46th weeks.

The dried food was prepared as described by Valenzano et al. [13]. Briefly, the blood worms were left to drip dry, and then 20 mg/ml SDS (purity > 98%; MANSITE BIO-TECHNOLOGY, China) dissolved in distilled water was added to the dry worms. The worms were equally divided into several pieces, each piece was added with the same SDS at a dose of 24 mg/kg of body weight and stored at 4°C for 2 h to soak. The worms with or without SDS were mixed with 5% gelatin and frozen at -20°C until use. Each fish was fed with one piece of the worms every day. The SDS was wrapped with gelatin and it did not dissolve in water easily, thus each fish could eat the whole piece of the worms and obtain the same quantity of SDS. The SDS-containing dried worms were named experimental diet, and the worms without SDS control diet. Totally, 190 individuals of 9-month-old male fish with average body weight of about 1.1 g/per fish were equally divided into two groups: one group of fish was fed with 40 mg experimental diet/g fish weight/day every day, with SDS at a dose of 24 mg/kg of body weight (The quantity of the diet was consumed by fish in a 15-min period, leaving no uneaten food); and the other group fed with 40 mg control diet/g fish weight/day every day. One hundred (50/each group) out of 190 fish were continuously cultured for assays of survivorship, body weight, and body length (Figure 9). The remaining 90 fish (45/each group) were cultured, and 12 fish were sampled from each group on 38th, 42th and 46th weeks (Figure 9), and anesthetized by cooling on ice. The 12 fish were divided into 3 sub-groups, each containing 4 individuals (Supplementary Table 1). The gills, muscles and fish body (FB) from the pectoral fin to tail fin containing only bones and red and white muscles (excluding head and internal organs) were dissected out of the fish of each sub-group, pooled and used for the following experiments.

### Survivor observation and body weight and length measurement

The health of each fish in the experimental (SDS) and control groups was surveilled every day, and the survival of fish was recorded until death of all the fish. Body weight and body length of fish were measured on 38th, 42th and 46th weeks respectively, as described by Markofsky and Perlmutter [41].

### Assay for histological marker

The gills were fixed in 4% formaldehyde in phosphate-buffered saline (PBS) at 4°C overnight, and precipitated in a 30% sucrose solution. They were embedded in optical cutting temperature compound, and sectioned at 10 μm thickness under -20°C (Leica, Germany). The auto-fluorescence of LF was observed under a fluorescence microscope with blue (450–490 nm) excitation light and 520 nm emission filter, and the fluorescent areas were quantified by Image J software [11].

### Assay for protein oxidation

The protein oxidation assay followed the method of Sohal et al [42]. A total of 1 gram of the muscle, including red and white ones, from 4 freshly killed fish was homogenized in 5 ml of 50 mM PBS (pH7.5) containing the protease inhibitors (leupeptin at 0.5 mg/ml, aprotinin at 0.5 mg/ml, pepstatin at 0.7 mg/ml, and phenylmethylsulfonyl fluoride [PMSF] at 40 mg/ml) using a Polytron and sonicator. The homogenates were centrifuged at 5000 g at 4°C for 10 min. The protein concentration of the supernatants was determined with a BCA Protein Assay Kit. Aliquots of 300 μl of the resulting supernatants with 2 to 2.5 mg of protein were treated with 300 μl of 10 mM 2, 4-dinitrophenylhydrazine (DNPH) dissolved in 2 M HCl or 2 M HCl alone (control). The mixtures were incubated at room temperature for 1 h (with agitation every 10 min), precipitated with 10% trichloroacetic acid (final concentration), and centrifuged at 12000 g at 4°C for 15 min. The pellets were washed three times with 1 ml of ethanol/ethyl acetate (vol/vol 1:1) and re-dissolved in 1 ml of 6 M guanidine in 10 mM PBS/trifluoroacetic acid (pH2.3). Any trace insoluble material was removed by centrifugation at 12000 g for 15 min. The difference in absorbance between the DNPH- and HCl-treated materials was determined at 366 nm, and expressed as nanomoles of carbonyl groups per milligram of protein, using the excitation coefficient of 22 mM^-1^cm^-1^ for aliphatic hydrazones.

### Assay for lipid peroxidation

To estimate lipid peroxidation levels, a lipid peroxidation MDA Assay Kit (Beyotime, China) was used to quantify the generation of MDA [43]. In brief, 1 gram of the fish body, FB (from 4 fish), was homogenized in 10 ml of 50 mM PBS (pH7.5) using a Polytron and sonicator. The homogenates were centrifuged at 5000 g at 4°C for 10 min, and the protein concentration of the supernatants was determined with a BCA Protein Assay Kit. Aliquots of 100 μl supernatants with 2 mg of protein were mixed with 200 μl of thiobarbituric acid (TBA) working solution in a test tube, and the mixtures incubated in a boiling water bath for 15 min. After cooling in tap water, the mixtures were centrifuged at 1000 g for 10 min, and their absorbances measured at 532 nm. The concentration of MDA was expressed as micromoles per gram of protein [44].

### Assay for CAT

CAT activity was estimated as described by Hsu et al. [27]. Aliquots of 1 g FB (from 4 fish) were homogenized in 10 ml of 50 mM PBS (pH7.5) using a Polytron and sonicator, and the homogenates prepared as above. The assay reaction consisted of 50 mM PBS (pH7.5), 100 μl of 30% hydrogen peroxide (H_2_O_2_), and the resulting supernatants in a total volume of 1 ml. The reaction was carried out at 25°C. A blank control was prepared with 900 μl of 50 mM PBS and 100 μl of 30% H_2_O_2_. The rates of absorbance change (ΔA/min) at 240 nm were recorded, which indicated the decomposition of H_2_O_2_. CAT activities were calculated using the molar extinction coefficient of H_2_O_2_ at 240 nm, 43.59 I/mol cm. Units of CAT were expressed as the amount of enzyme that decomposes 1 μmol of H_2_O_2_ per min at 25°C. The specific activity was expressed in terms of micromoles per minute per milligram of protein.

### Assay for GPX

GPX activity assay was measured by the method of Hsu et al. [27]. Briefly, aliquots of 1 g FB (from 4 fish) were homogenized in 10 ml of 50 mM Tris-HCl buffer (pH7.5) containing the protease inhibitors (0.5 μg/ml leupeptin, 0.5 μg/ml aprotinin, 0.7 μg/ml pepstatin, 40 μg/ml PMSF) using a Polytron and sonicator, and the homogenates prepared as above. An aliquot of 10 μl supernatant with 0.5 mg of protein was mixed with 10 μl of GPX working solution (5 mM nicotinamide adenine dinucleotide phosphate [NADPH], 42 mM reduced glutathione, and 20 U glutathione reductase), and then with 176 μl GPX assay buffer (50 mM Tris-HCl containing 2 mM EDTA, pH7.5). All the solutions were pre-incubated at 25°C before mixing. The reaction was initiated by adding 4 μl of 15 mM tert-butyl-hydroperoxide (t-Bu-OOH; Beyotime, China) in Milli-Q-grade water. The absorbance (optical density [OD]) was recorded at 340 nm every 30 sec. One unit of GPX activity was defined as the amount of enzyme that hydrolyzes 1.0 μmol of NADPH into NADP^+^ per minute under the conditions described [45].

### Assay for total SOD

The assay of SOD was based on the reduction of nitroblue tetrazolium (NBT) to water-insoluble blue formazan [46]. Briefly, 1 g of FB (from 4 fish) was homogenized in 10 ml of 50 mM PBS (pH7.5) using a Polytron and sonicator. The homogenate was centrifuged at 5000 g at 4°C for 10 min, and the supernatant was pooled. Total SOD activity was assayed according to the instructions of the SOD Assay Kit (Beyotime, China). The rate of NBT reduction was monitored at 560 nm at 25°C. One unit of SOD was defined as the amount of protein that resulted in 50% inhibition of the rate of NBT reduction.

### Gene fragment cloning and quantitative real-time PCR (qRT-PCR)

The muscles dissected out of freshly killed *N. guentheri* were ground in RNAiso Plus (TaKaRa) and kept at -70°C until use. Total RNAs were isolated from the frozen samples according to the manufacturer’s instructions. After digestion with recombinant DNase I (RNase free; TaKaRa) to eliminate the genomic contamination, cDNAs were synthesized with a reverse transcription kit (TaKaRa) with oligo (dT) primer. The reaction was carried out at 42°C for 1 h and inactivated at 75°C for 15 min. The cDNAs were stored at –20°C till used [43].

For cloning of the gene *p66shc*, a pair of gene-specific primers PS and PA (Table 1) was designed on the basis of *p66shc* gene sequence using the Primer Premier program (version 5.0), and used for PCR. The amplification products were cloned into a pGEM-T Easy vector (TIANGEN) following the manufacturer’s instructions and transformed into Trans5α bacteria (TRANSGEN). The positive clones were selected and sequenced using an ABI PRISM 3730 DNA sequencer. The sequences were compared with related sequences available in GenBank using BLASTx. The sequence of *p66shc* we obtained was deposited in GenBank and the accession number is: MF346704. To compare the similarity of *N. guentheri* P66shc with mammalian ortholog P66SHC, homology searches in the GenBank™ database were carried out using the BLAST server, and multiple alignments of the protein sequences generated using the Clustal W program [47] within MegAlign of the DNASTAR software package.

**Table 1:** Sequences of the primers used in this study

Primers	Sequences (5′-3′)
For p66shc cloning	
PS (sense)	ATGGAGCTCATGCAGAAAACCAAGT
PA (antisense)	TTAGGCCTTGCGCTCCACTGGCTGT
For qRT-PCR	
qPS (sense)	CCACCTTATGCTCCTTCTTCCCCAG
qPA (antisense)	CTGGGACTGCTGAGACCTGTTTGAG
*β*-actinS (sense)	CACCTTCTACAATGAGCTCCGT
*β*-actinA (antisense)	GCAGGAGTGTTGAAGGTCTCAA
For expression in eukaryotic vector	
ePS (sense)	GCC AAGCTT ATGGAGCTCATGCAGAAAACCAAGT
ePA (antisense)	CCG GAATTC GGCCTTGCGCTCCACTGGCTGT

Sequences of the primers used in this study. The cutting sites of restriction enzyme were underlined

To detect the expression profiles of *p66shc* in the muscles from fish fed with the different diets, qRT-PCR was performed using the first-strand cDNAs as template, which was reverse-transcribed from the total RNAs extracted from the muscles. One pair of primers specific of *p66shc* (qPS and qPA; Table 1) was designed using the Primer Premier program (version 5.0). The reaction mixture (final volume 20 μl) consisted of 10 μl of SYBR Premix Ex Taq (Tli RNaseH Plus), 0.4 μl ROX Reference Dye II, 0.5 μl of template, and 200 nM each of sense and antisense primers. The *β-actin* gene was chosen as the reference for internal standardization. All the qRT-PCR experiments were conducted in triplicate. The amplification was performed on an ABI 7500 Real-Time PCR System (Applied Biosystems) at 95°C for 15 sec, followed by 40 cycles of 95°C for 5 sec, 60°C for 15 sec, and 72°C for 35 sec. The expression level of *p66shc* relative to that of the *β-actin* gene was calculated by the comparative threshold cycle (CT) method (2^-ΔΔCT^) [48].

### Construction of eukaryotic expression vector

For eukaryotic expression vector construction, the open reading frame of p66shc was amplified by PCR using the primers ePS with *Hind* III site and ePA with *EcoR* I site (Table 1), and sub-cloned into the plasmid expression vector pcDNA3.1/V5/His (Invitrogen), and the recombinant was designated *pcDNA3.1/p66shc/V5/His*.

### Cell culture and transfection

HEK 293T cells (gifts of Jianfeng Zhou, Laboratory of Molecular Medicine, School of Medicine and Pharmacy, Ocean University of China) were cultured at 37°C with 5% CO_2_ in Dulbecco’ modified Eagle medium (Invitrogen) supplemented with 10% (v/v) fetal bovine serum (Invitrogen). They were seeded in 6-well plates and transfected with the different plasmids using Lipofectamine 2000 Reagent (Invitrogen) [49]. Briefly, *pcDNA3.1/p66shc/V5/His* plasmid was mixed with Lipofectamine 2000, and the mixtures were added into HEK 293T cells and incubated at 37°C for 24 h. Total Protein Extraction Kit (TRANSGEN, China) was used for preparation of total protein extracts from HEK 293T cells according to the manufacture’s protocol. To further determine the expression of P66SHC, the proteins were subjected to Western blotting analysis. For control, HEK 293T cells were either transfected with pcDNA3.1/V5/His or non-transfected at all, and then processed similarly.

### Western blotting

A total of 1 g of the muscle from 4 freshly killed fish was homogenized in 5 ml of 50 mM PBS (pH7.5) containing the protease inhibitors, and centrifuged at 5000 g at 4°C for 10 min. The proteins from *N. guentheri* muscles as well as the proteins extracted from HEK 293T cells were subjected to electrophoresis on a 12% sodium dodecyl sulfate polyacrylamide gel electrophoresis (SDS-PAGE) gel (Each lane of the gel was loaded with 20 μg of protein). The proteins on the gel were transferred to polyvinylidene difluoride (PVDF) membranes, which were blocked with 4% bovine serum albumin (BSA) in 10 mM PBS (pH7.5) at room temperature for 2 h. They were incubated with the following respective antibodies: anti-SHC polyclonal antibody (1:1000, ab155170; Abcam, UK), anti-His tag monoclonal antibody (1: 5000, CW0082B; CWBIO, China) and anti-Actin antibody (1:2000, bs-0061R; Bioss, China) at 4°C overnight. After washing in 10 mM PBS (pH7.5) containing 0.1% Tween-20, the membranes were then probed with a secondary horseradish peroxidase (HRP)-labeled antibody (1:3000) at room temperature for 3 h. The bands were visualized by ECL Western blotting substrate (Thermo Fisher Scientific, USA) and analyzed using software Image J [50].

### Assay for ROS

The quantitative determination of ROS was performed using dichloro-dihydro-fluorescein diacetate (DCFH-DA) as described by Dong et al. [51]. Aliquots of 1 g of the muscles (from 4 fish) were homogenized in 2.5 ml of 50 mM PBS (pH7.5), centrifuged at 5000 g at 4°C for 30 min, and the supernatants collected. The protein concentration of the supernatants was determined with a BCA Protein Assay Kit (CWBIO, China). A total of 1.8 ml of the supernatant with 1.8 mg protein was mixed with 1.8 μl of 10 mM DCFH-DA (Beyotime, China), and the mixture incubated at 37°C for 30 min. The fluorescence intensity was measured at excitation and emission wavelengths of 488 nm and 525 nm.

### Statistical analysis

Except fish culture, all the experiments were repeated at least three times. The single values for each replicas of the data were shown in Supplementary Table 2. Statistical analysis was performed using GraphPad Prism 5 program. All the data were expressed as mean ± standard deviation (SD). The data regarding survival curves were subjected to the log-rank test. One-way ANOVA was used for the analysis of difference. Two-way ANOVA was used to estimate the interaction between two factors (age×treatment). A difference at *p* < 0.05 was considered significant.

## SUPPLEMENTARY MATERIALS FIGURES AND TABLES




