# Supplementary material for: Time-dependent effects of late-onset dietary intake of salidroside on lifespan and age-related biomarkers of the annual fish Nothobranchius guentheri
Source: Oncotarget. 2018 Jan 4;9(19):14882–94. doi: 10.18632/oncotarget.23957 (PMC5871084; doi:10.18632/oncotarget.23957)
Supplement: Supplementary file 1 [file oncotarget-09-14882-s001.pdf]

Time-dependent effects of late-onset dietary intake of salidroside on lifespan and age-related biomarkers of the annual fish

SUPPLEMENTARY MATERIALS

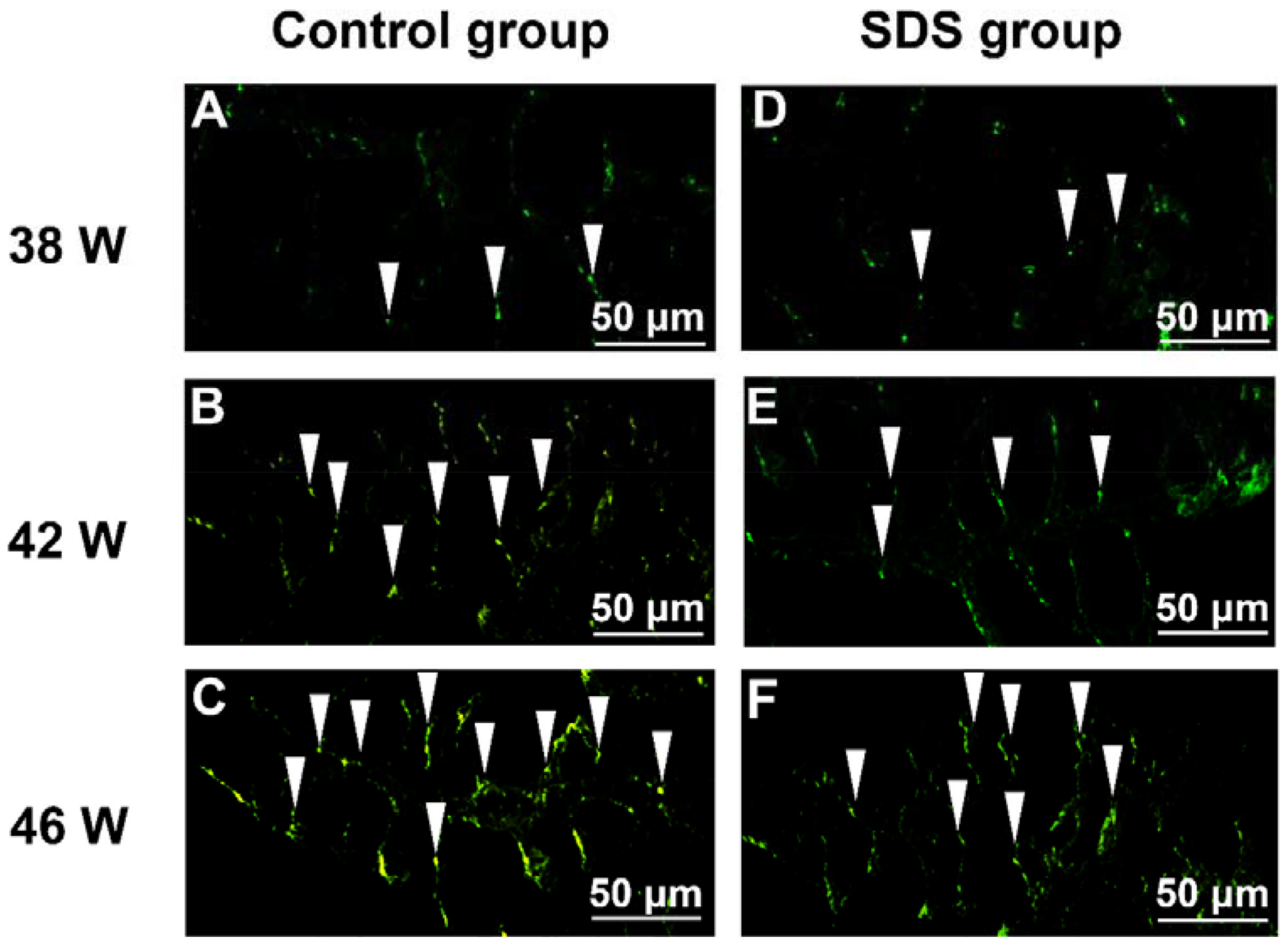

Supplementary Figure 1: Changes in the histological marker LF in 38-, 42- and 46-week-old N. guentheri. Supplementary1 Changes of the histological marker LF in 38-, 42- and 46-week-old N. guentheri. (**A–C**), Accumulation of LF in the gills of N. guentheri in control group; (**D–F**), Accumulation of LF in the gills of N. guentheri in SDS group. Bar is 50 μm. w, week.

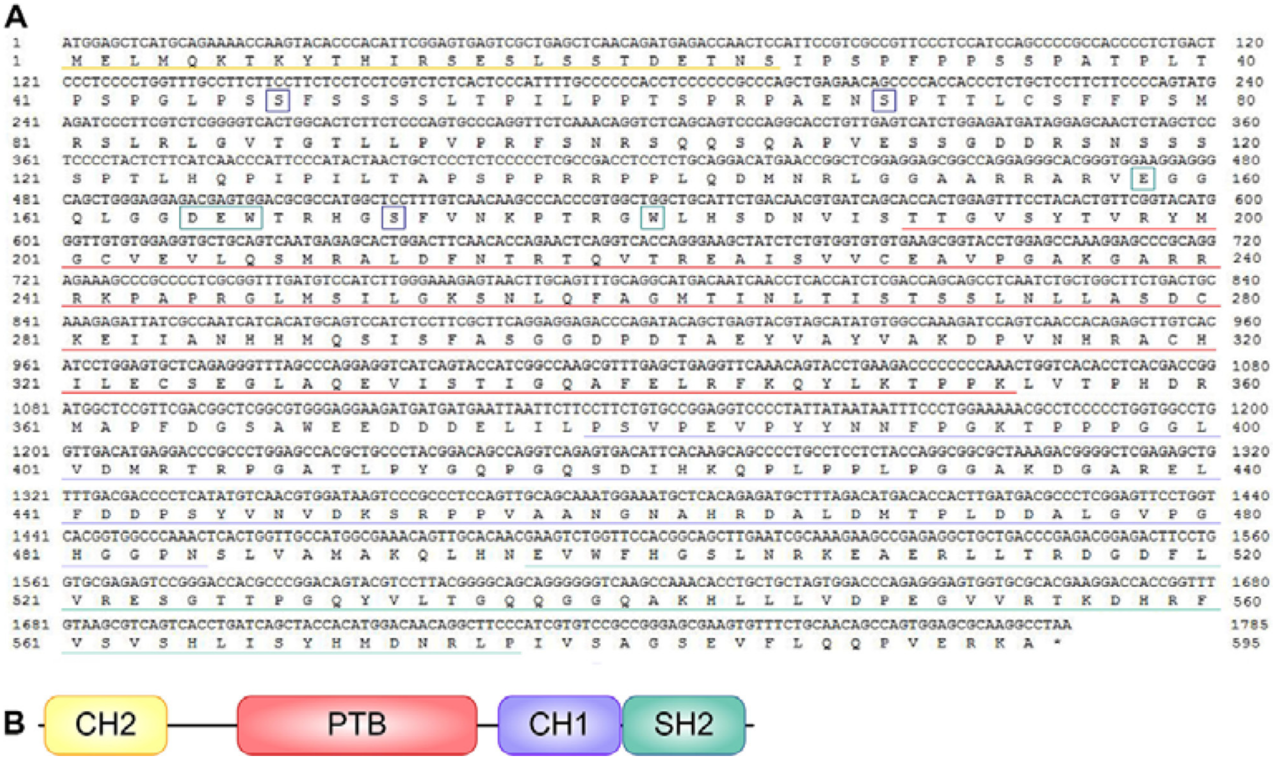

Supplementary Figure 2: The sequence and domains of N. guentheri P66shc. (**A**) The derived amino acid sequence is shown underneath the nucleotide sequence using single-letter codes. The stop codon is indicated by an asterisk. Four domains of N. guentheri P66shc are indicated by yellow, red, violet and green underlines. Three conserved S residues are shown by violet boxes, and a conserved cytochrome c binding domain constituted of E, D and W residues are shown by green boxes. (**B**) Four domains of N. guentheri P66shc.

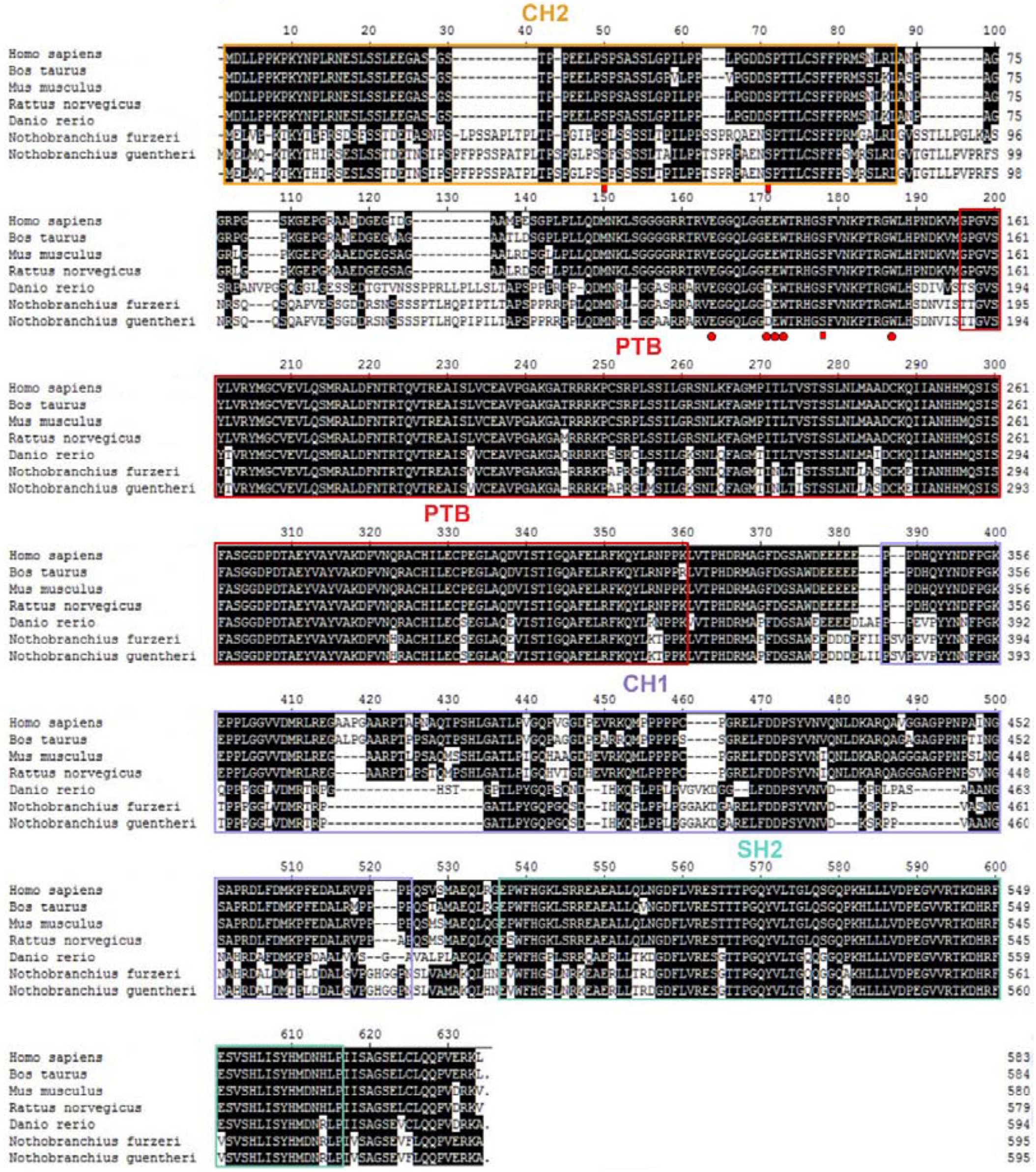

Supplementary Figure 3: Multiple alignment of N. guentheri P66shc and other known P66SHC amino acid sequences using the Clustal W program within the MegAlign of the DNASTAR software package (version 5.0). Shaded (with solid black) residues are the amino acids that match the consensus. The CH2, PTB, CH1 and SH2 domain are indicated by yellow, red, violet and green boxes. Conserved S residues are indicated by solid red boxes, cytochrome c binding domain is constituted of E, D, W residues which are indicated by solid red dots. Amino acid numbering is indicated on the right.

Percent Identity

Divergence	1	2	3	4	5	6	7		
1		95.0	95.0	94.6	74.5	71.3	71.5	1	*Homo sapiens*
2	5.2		94.0	93.3	73.5	69.8	70.1	2	*Bos taurus*
3	5.2	6.3		98.6	73.8	70.0	70.3	3	*Mus musculus*
4	5.6	7.1	1.4		73.6	69.8	70.0	4	*Rattus norvegicus*
5	31.2	32.8	32.2	32.6		81.3	81.7	5	*Danio reio*
6	36.2	38.5	38.2	38.5	21.6		99.2	6	*Nothobranchius furzeri*
7	35.9	38.2	37.9	38.2	21.1	0.8		7	*Nothobranchius guentheri*
	1	2	3	4	5	6	7		

Supplementary Figure 4: Amino acid sequence identity of N. guentheri P66shc with other species P66SHC proteins. The amino acid identity was calculated using the Clustal W program within the MegAlign of the DNASTAR software package (version 5.0). The sequence comparison showed that N. guentheri P66shc shares 70% to 71.5% identity to mammalian P66SHC..

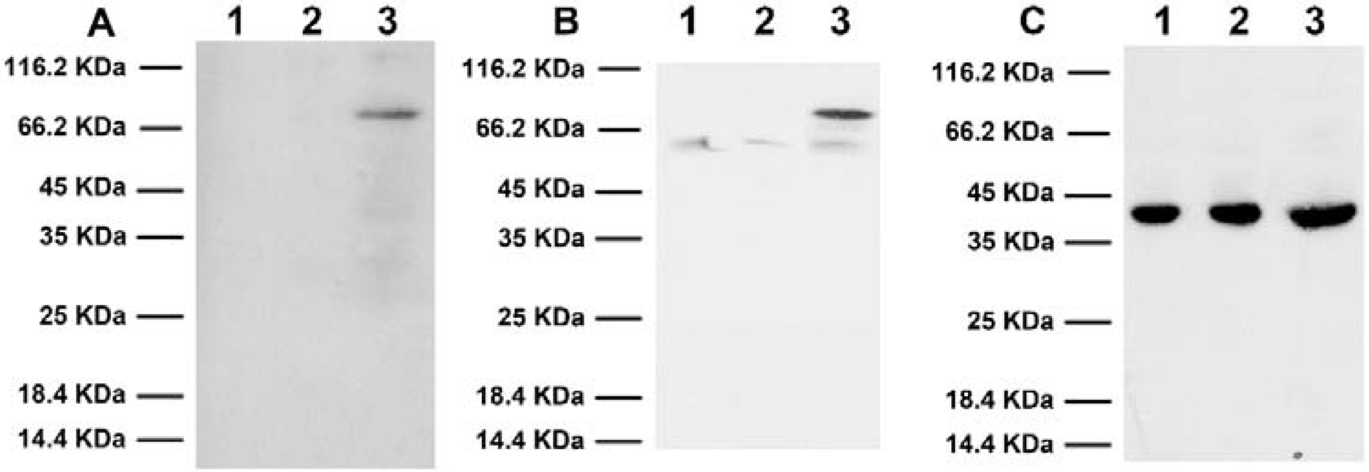

Supplementary Figure 5: Western blotting of P66SHC and Actin in HEK 293T cells. (**A**) anti-His tag antibody reacted with proteins of HEK 293T cells; (**B**) anti-SHC antibody reacted with proteins of HEK 293T cells; (**C**) anti-Actin antibody reacted with proteins of HEK 293T cells. Lane 1, total proteins of HEK 293T cells; Lane 2, total proteins of HEK 293T cells transfected with pcDNA3.1/V5/His; Lane 3, total proteins of HEK 293T cells transfected with pcDNA3.1/p66shc/V5/His.

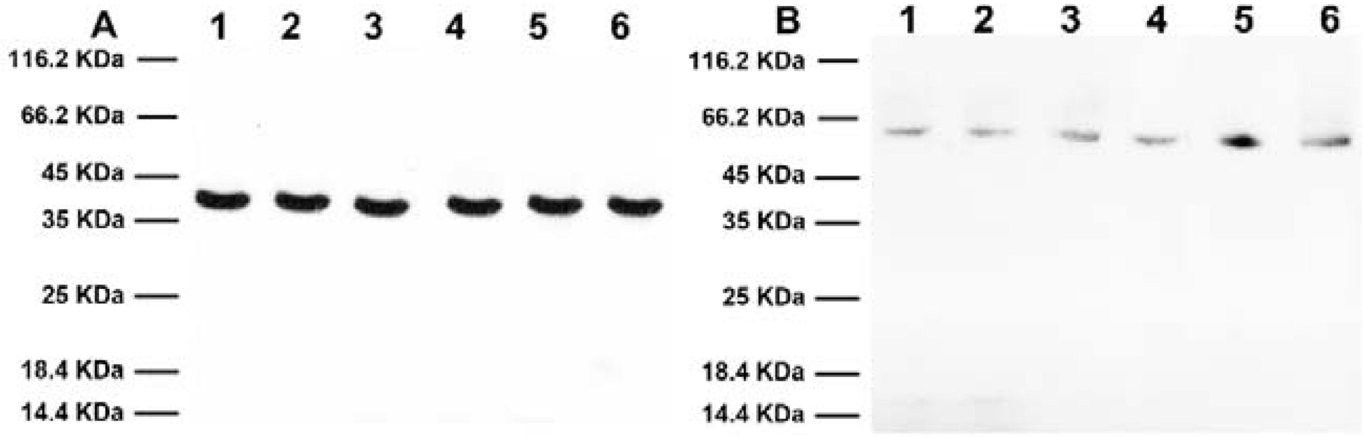

Supplementary Figure 6: Western blotting of Actin and P66shc in muscles of N. guentheri. There was only a band about 42 kDa and 64 kDa in the entire gel, respectively. (**A**) Actin protein in N. guentheri muscles; (**B**) P66shc protein in N. guentheri muscles; Lanes 1, 3 and 5, protein extracts from 38, 42 and 46-week-old N. guentheri muscles in control group; Lanes 2, 4 and 6, protein extracts from 38, 42 and 46-week-old N. guentheri muscles in SDS group.

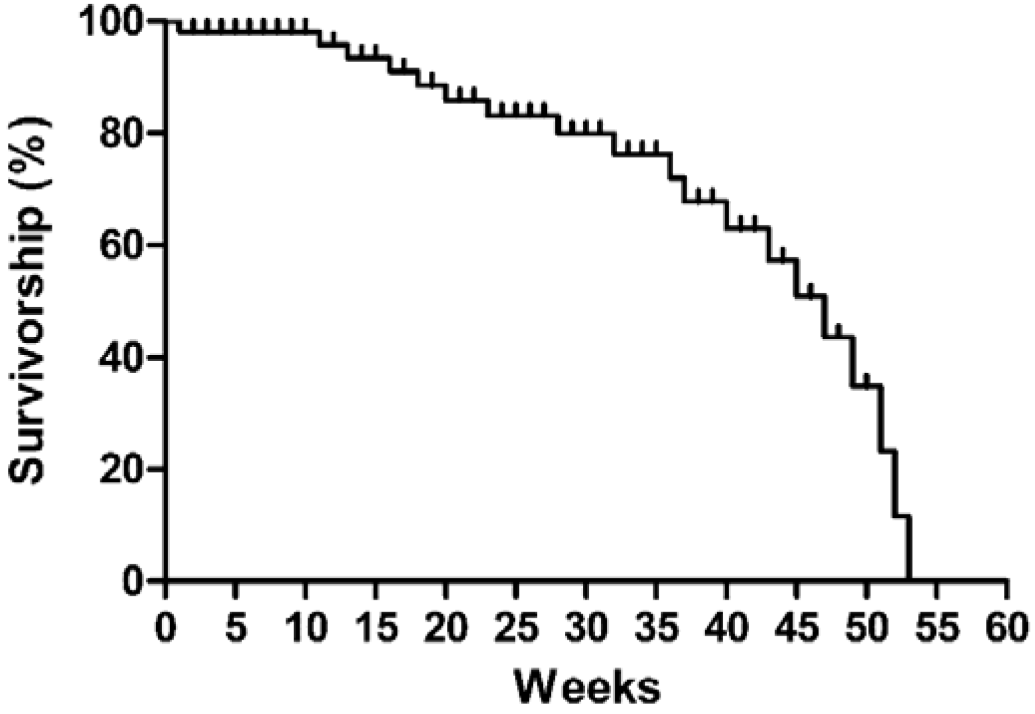

Supplementary Figure 7: Survival curve of N. guentheri.

Supplementary Table 1: The number of fish used for assay of aging makers at each age in each group

Group	Markers			Count		
	Protein oxidation and lipid peroxidation	LF, CAT and GPX	SOD, p66shc/P66SHC and ROS	Each stage	3 stages	Total
Control	4	4	4	12	36	72
SDS	4	4	4	12	36	

Supplementary Table 2: The single values for each replicas of the data in the text, we have clarified this in the text. See Supplementary_Table_2
