# Supplementary material for: Time-dependent effects of late-onset dietary intake of salidroside on lifespan and age-related biomarkers of the annual fish Nothobranchius guentheri
Source: Oncotarget. 2018 Jan 4;9(19):14882–94. doi: 10.18632/oncotarget.23957 (PMC5871084; doi:10.18632/oncotarget.23957)
Supplement: Supplementary file 2 [file oncotarget-09-14882-s002.docx]

**Supplementary Table 2:**

**a**. Body weight of male *N. guentheri*.

| Age | Body weight (g) | | | | | | | |
| --- | --- | --- | --- | --- | --- | --- | --- | --- |
|  | Control group | | | mean | SDS group | | | mean |
| 38 w | 1.0540 | 1.1444 | 1.1855 | 1.128  ±0.0673 | 1.0244 | 1.1344 | 1.2024 | 1.120  ±0.0898 |
| 42 w | 1.0723 | 1.1556 | 1.2144 | 1.147  ±0.0714 | 1.0449 | 1.1449 | 1.2534 | 1.148  ±0.1043 |
| 46 w | 1.1040 | 1.1742 | 1.2432 | 1.174  ±0.0696 | 1.0634 | 1.1552 | 1.3040 | 1.174  ±0.1214 |

**b.** Body length of male *N. guentheri*.

| Age | Body length (cm) | | | | | | | |
| --- | --- | --- | --- | --- | --- | --- | --- | --- |
|  | Control group | | | mean | SDS group | | | mean |
| 38 w | 4.1534 | 4.0241 | 4.2758 | 4.151  ±0.1259 | 4.1234 | 3.9124 | 4.2734 | 4.083  ±0.1545 |
| 42 w | 4.2314 | 4.1512 | 4.3521 | 4.245  ±0.1011 | 4.1813 | 4.0910 | 4.3320 | 4.169  ±0.0720 |
| 46 w | 4.3316 | 4.2210 | 4.4210 | 4.325  ±0.1002 | 4.2821 | 4.1500 | 4.4084 | 4.247  ±0.0849 |

**c.** Changes in the histological marker LF in 38-, 42- and 46-week-old *N. guentheri*.

| Age | LF (Threshold of area of LF (%)) | | | | | | | |
| --- | --- | --- | --- | --- | --- | --- | --- | --- |
|  | Control group | | | mean | SDS group | | | mean |
| 38 w | 4.1627 | 4.2406 | 4.1008 | 4.168  ±0.0701 | 3.5119 | 3.5349 | 3.5284 | 3.525  ±0.0118 |
| 42 w | 7.5930 | 7.930 | 7.6318 | 7.718  ±0.1846 | 6.1823 | 6.4929 | 6.3679 | 6.348  ±0.1563 |
| 46 w | 13.3123 | 10.906 | 14.3469 | 12.86  ±1.765 | 8.8006 | 7.4895 | 8.2798 | 8.19  ±0.6601 |

**d.** Levels of protein oxidation in 38-, 42- and 46-week-old *N. guentheri*.

| Age | Carbonyl-group content (nmol/mg protein) | | | | | | | |
| --- | --- | --- | --- | --- | --- | --- | --- | --- |
|  | Control group | | | mean | SDS group | | | mean |
| 38 w | 21.2121 | 21.4255 | 21.8374 | 21.49  ±0.3178 | 21.0594 | 21.1342 | 21.4325 | 21.21  ±0.1974 |
| 42 w | 24.4564 | 23.5656 | 24.5640 | 24.20  ±0.5480 | 22.3430 | 21.9543 | 22.7316 | 22.34  ±0.3886 |
| 46 w | 27.8593 | 26.8598 | 28.0325 | 27.55  ±0.6153 | 24.5325 | 24.3436 | 23.1443 | 24.01  ±0.7529 |

**e.**Levels of lipid peroxidation in 38-, 42- and 46-week-old *N. guentheri*.

| Age | MDA (μM/mg protein) | | | | | | | |
| --- | --- | --- | --- | --- | --- | --- | --- | --- |
|  | Control group | | | mean | SDS group | | | mean |
| 38 w | 3.4229 | 3.3975 | 3.4723 | 3.431  ±0.0384 | 3.3930 | 3.3925 | 3.4170 | 3.401  ±0.0140 |
| 42 w | 3.6175 | 3.6587 | 3.6853 | 3.654  ±0.0341 | 3.4342 | 3.4979 | 3.4757 | 3.469  ±0.0323 |
| 46 w | 3.8542 | 3.9535 | 3.9501 | 3.919  ±0.0564 | 3.6442 | 3.7474 | 3.6381 | 3.677  ±0.0614 |

**f**. Changes in activities of CAT in 38-, 42- and 46-week-old *N. guentheri*.

| Age | CAT (μM/min/mg protein) | | | | | | | |
| --- | --- | --- | --- | --- | --- | --- | --- | --- |
|  | Control group | | | mean | SDS group | | | mean |
| 38 w | 3.7251 | 3.6471 | 3.6030 | 3.6580±0.0618 | 3.7090 | 3.6534 | 3.6006 | 3.6540±0.0542 |
| 42 w | 3.0323 | 2.9404 | 2.8773 | 2.9500±0.0779 | 3.2232 | 3.1510 | 3.0234 | 3.1330±0.1012 |
| 46 w | 2.4109 | 2.4908 | 2.5402 | 2.4810±0.0653 | 2.7528 | 2.6501 | 2.6913 | 2.6980±0.0517 |

**g.** Changes in activities of GPX in 38-, 42- and 46-week-old *N. guentheri*.

| Age | GPX (nM/min/mg protein) | | | | | | | |
| --- | --- | --- | --- | --- | --- | --- | --- | --- |
|  | Control group | | | mean | SDS group | | | mean |
| 38 w | 7.3235 | 6.3235 | 8.2025 | \| 7.283 \| \| --- \| \| ±0.9401 \| | 7.2635 | 6.3517 | 8.2524 | \| 7.289 \| \| --- \| \| ±0.9506 \| |
| 42 w | 6.4660 | 5.6560 | 7.4656 | \| 6.529 \| \| --- \| \| ±0.9065 \| | 6.8563 | 5.8970 | 7.7545 | \| 6.836 \| \| --- \| \| ±0.9289 \| |
| 46 w | 5.7350 | 4.8435 | 6.6250 | \| 5.735 \| \| --- \| \| ±0.8908 \| | 6.3792 | 5.3535 | 7.2526 | \| 6.320 \| \| --- \| \| ±0.9506 \| |

**h.** Changes in activities of SOD in 38-, 42- and 46-week-old *N. guentheri*.

| Age | SOD (U/mg protein) | | | | | | | |
| --- | --- | --- | --- | --- | --- | --- | --- | --- |
|  | Control group | | | mean | SDS group | | | mean |
| 38 w | 27.3000 | 26.9700 | 21.7100 | \| 25.33 \| \| --- \| \| ±3.136 \| | 27.5900 | 27.0200 | 22.0500 | \| 25.55 \| \| --- \| \| ±3.047 \| |
| 42 w | 21.5650 | 21.5654 | 18.4530 | \| 20.53 \| \| --- \| \| ±1.797 \| | 22.4640 | 22.6342 | 19.3530 | \| 21.48 \| \| --- \| \| ±1.847 \| |
| 46 w | 15.7693 | 16.0453 | 15.8651 | \| 15.89 \| \| --- \| \| ±0.1401 \| | 17.7843 | 17.8595 | 17.4349 | \| 17.69 \| \| --- \| \| ±0.2266 \| |

**i.** ROS levels in the muscles of 38-, 42- and 46-week-old *N. guentheri*.

| Age | ROS (fluorescence intensity/mg protein) | | | | | | | |
| --- | --- | --- | --- | --- | --- | --- | --- | --- |
|  | Control group | | | mean | SDS group | | | mean |
| 38 w | 401.324 | 414.342 | 432.414 | 416.0  ±15.61 | 390.414 | 404.421 | 422.324 | 405.7  ±15.99 |
| 42 w | 442.330 | 451.420 | 463.420 | 452.4  ±10.58 | 422.424 | 437.420 | 443.424 | 434.4  ±10.82 |
| 46 w | 495.244 | 513.122 | 522.535 | 510.3  ±13.86 | 465.420 | 487.420 | 504.530 | 485.8  ±19.55 |
